# The role of ego integrity and despair in older adults’ well-being during the COVID-19 crisis: the mediating role of need-based experiences

**DOI:** 10.1007/s10433-021-00610-0

**Published:** 2021-02-28

**Authors:** Jolene van der Kaap-Deeder, Branko Vermote, Joachim Waterschoot, Bart Soenens, Sofie Morbée, Maarten Vansteenkiste

**Affiliations:** 1 Department of Psychology, Norwegian University of Science and Technology (NTNU), 7491 Dragvoll Trondheim, Norway; 2 Department of Developmental, Social, and Personality Psychology, Ghent University, Ghent, Belgium

**Keywords:** Older adults, Ego integrity, Despair, Basic psychological needs, Self-determination theory, COVID-19

## Abstract

The COVID-19 pandemic has affected people across the world, with important heterogeneity among older adults in how they respond to the challenges associated with this crisis. Relying on a cross-fertilization between Erikson’s personality theory and self-determination theory, this study aimed to examine possible sources of resilience (i.e., ego integrity and need satisfaction) and vulnerability (i.e., despair) in older adults’ (mal) adjustment, thereby additionally considering the role of multiple risk and protective factors (e.g., gender and marital status). During the second month of the lockdown period in Belgium, 693 older adults (*M*_age_ = 70.06, *SD* = 4.48, range: 65–89 years, 62.1% female) filled out online questionnaires concerning the study variables, while also completing assessments of several important sociodemographic factors. Structural equation modeling suggested that both ego integrity and despair related to indicators of well-being and psychological distress through experienced need satisfaction. Additionally, we found several factors to protect (e.g., higher perceived income) or diminish (e.g., being widowed) older adults’ well-being during these challenging times, with little evidence for a moderating role of these factors in associations between the psychological variables. Theoretical and practical implications of these results are discussed.

## Introduction

In 2020, the lives of people across the world were impacted heavily by the COVID-19 pandemic. The virus not only represents a direct health threat but also the public measures taken to mitigate the transmission of the virus further interrupted people’s normal, daily routines in an intrusive manner. Some initial research indeed indicates that older adults have been especially vulnerable during this pandemic, as indicated by higher mortality risk (WHO 2020), increased social isolation, delay of medical treatment, difficulties to engage in daily activities (e.g., not being able to go grocery shopping) (Miller 2020), and reduced physical activity (Di Santo et al. 2020). Other research, however, showed an opposite pattern, with late adults reporting less psychological distress compared to other age-groups (e.g.,Bäuerle et al. 2020; Losada-Baltar et al. 2020) and experiencing similar or even more well-being compared to before the COVID-19 crisis (Kivi et al. 2020). In light of these findings, it is critical to identify the sources underlying older adults’ psychological functioning during this pandemic, possibly explaining the heterogeneity among this age-group. Based on self-determination theory (SDT; Deci and Ryan 2000; Ryan and Deci 2017) and Erikson's theory of psychosocial development (Erikson 1963), the overall aim of this study was to examine whether older adults’ successful resolution of the psychosocial crisis of ego integrity versus despair (i.e., experiencing a sense of coherence versus regret with respect to one’s life) would relate to their well-being during the COVID-19 crisis via levels of need satisfaction, with several risk (e.g., illness) and protective factors (e.g., higher income) possibly moderating these relations.

### The importance of ego integrity and despair for older adults’ well-being

Erikson (1963) stated that personality develops across the lifespan through eight sequential psychosocial crises, where the successful resolution of each crisis fosters virtues (e.g., hope, fidelity, or, in later life, wisdom) and lays the foundation of success during the next crisis. From the age of about 65 years, individuals face the last of eight psychosocial crises, namely a crisis concerning achieving a sense of ego integrity while avoiding despair. During this crisis, elderly reflect on their life in an attempt to unify past events into a meaningful “life puzzle” and they come to terms with past negative events. When successful, individuals will experience a sense of ego integrity, where they can accept past events, see their life in a coherent perspective, and regard death as a natural and integral part of life. Despair, on the other hand, is concerned with difficulties in accepting and finding wholeness in one’s own life path and often comes with high levels of regret. Although most studies focused solely on ego integrity, several studies have shown that ego integrity and despair are not mere opposites. When elderly do not feel desperate about life choices that were made, they have not necessarily achieved a sense of ego integrity. Demonstrating the distinction between both orientations, they were found to develop differently across time, with ego-integrity increasing particularly between early midlife (age 43) and late life (age 72) and with despair increasing from age 43 to age 53 and then decreasing until the age of 72 (Newton et al. 2019).

Although the crisis of ego integrity and despair becomes especially salient during late adulthood, it can also surface when older individuals are confronted with challenging contexts and events, such as the loss of loved ones and illness (Kivnick and Wells 2014). The COVID-19 crisis posed many of these psychological challenges and perhaps even raised fundamental existential questions among many people. Confronted with an increased risk for illness and death, people may wonder more often about the meaning of their past life and about their current life structure. Depending on whether they address the existential questions with a sense of ego integrity or with a sense of despair, people are likely to experience the COVID-19 crisis very differently, with those having achieved a sense of ego integrity being better capable to act in a resilient way and those being high in despair instead being more vulnerable for maladjustment. Congruent with this reasoning, previous studies found ego integrity to relate to higher levels of mental health (e.g., life satisfaction) and lower levels of psychological distress (e.g., depressive symptoms), whereas despair shows an opposite pattern of associations (e.g., Derdaele et al. 2019; Lamers et al. 2011; Van Hiel and Vansteenkiste 2009).

### The explanatory role of need-based experiences

Recent research has begun to examine mechanisms that may underlie associations between ego-integrity, despair, and late adults’ mental health. On the basis of SDT, Van der Kaap-Deeder et al. (2020) considered the role of individuals’ need-based experiences. Within SDT, it is postulated that the satisfaction of three innate psychological needs is essential for individuals’ thriving and well-being (Ryan and Deci 2017; Vansteenkiste et al. 2020). First, the need for autonomy denotes experiencing a sense of personal freedom and choice. This need is satisfied, for instance, when individuals feel that their actions are congruent with who they are, whereas feelings of pressure and coercion are indicative of autonomy frustration. Second, the need for competence refers to the experience of mastery and effectiveness. Being able to successfully pursue personally important goals signifies competence satisfaction. In contrast, when feeling overwhelmed by situational demands and feeling like a failure, individuals experience a high level of competence frustration. Third, the need for relatedness entails experiencing a sense of belonging and mutual care. This need is satisfied, for instance, when individuals feel connected to and understood by important others, whereas feelings of isolation and social exclusion are suggestive of relatedness frustration. Due to the COVID-19 pandemic, individuals are likely to experience difficulties in getting these needs met, thereby experiencing for instance restriction of choice (autonomy frustration), solitude and social alienation (relatedness frustration), and inadequacy in pursuing important goals (competence frustration).

A vast number of studies, mostly focusing on children, adolescents, and (young) adults, have documented the beneficial effects of need satisfaction and the detrimental effects of need frustration for individuals’ mental health (see, for an overview, Ryan and Deci 2017; Vansteenkiste and Ryan 2013). However, research increasingly demonstrates the importance of these psychological needs for the well-being of older adults as well (e.g., Custers et al. 2013). Indeed, Tang et al. (2020) showed in a meta-analysis of 17 studies among older adults that need satisfaction was positively associated with indicators of well-being (such as life satisfaction) and negatively associated with indicators of psychological distress (such as depression). Longitudinal studies even showed that need satisfaction is predictive of better psychological adjustment to retirement within a six-year period (Houlfort et al. 2015) and of increased psychological adjustment over a one-year period among nursing home residents (Philippe and Vallerand 2008). Examining the unique role of the psychological needs, Neubauer et al. (2017) showed that competence, but not autonomy, related to subjective well-being among individuals aged over 87 years at the within-person level, whereas at the between-person level both autonomy and competence related to negative affect and only competence was associated with positive affect and life satisfaction. Further, Henning et al. (2019) found all three psychological needs to uniquely predict subjective well-being among late adults at the within-person level, but at the between-person level only autonomy and relatedness (but not competence) predicted well-being.

Conceptually, ego integrity is likely to foster experiences of need satisfaction and despair is likely to engender need frustration (Van der Kaap-Deeder et al. 2020). That is, individuals who regard their lives as meaningful and have come to terms with difficult past events are more likely to experience feelings of volition, competence, and connectedness with important others. Several processes can account for these associations, including a more positive appraisal of potentially need-satisfying events among people high on ego-integrity and an inclination to proactively seek more need-satisfying contexts and activities. In line with this reasoning, James and Zarrett (2006) showed that ego integrity in women aged over 70 was positively related to feelings of autonomy, mastery, and positive relationships with others (see Ferrand et al. 2014 for similar evidence).

### Risk factors during the COVID-19 crisis

Although some older adults have been particularly hard hit by the COVID-19 pandemic (Miller 2020), certain factors are likely to have increased or decreased the negative impact of the pandemic among this population. Specifically, older adults with specific health risks such as chronic respiratory issues are more likely to be affected by the COVID-19 pandemic (Cohen and Tavares 2020), perhaps compromising their well-being. Research also showed that older women display lower subjective well-being compared to older men (Lukaschek et al. 2017). On the positive side, having a higher education could be a protective factor, with previous studies showing university-educated older adults to report a higher level of ego integrity (Solcova et al. 2020) and well-being (Wiesmann and Hannich 2008). Using electronic devices such as smartphones to connect with others might also enable older adults to cope better with the situational demands of the pandemic. Smartphone use (but not smartphone proficiency) has been linked to decreased loneliness and increased ego integrity (Kim et al. 2020). Income, social status, and family relationships (e.g., with their own children) have also been found to affect older adults’ well-being and ego integrity (e.g., Hannah et al. 1996; James and Zarrett 2006) and could provide a financial or social buffer against the hardships imposed by the COVID-19 crisis.

### The present research

**Fig. 1:**
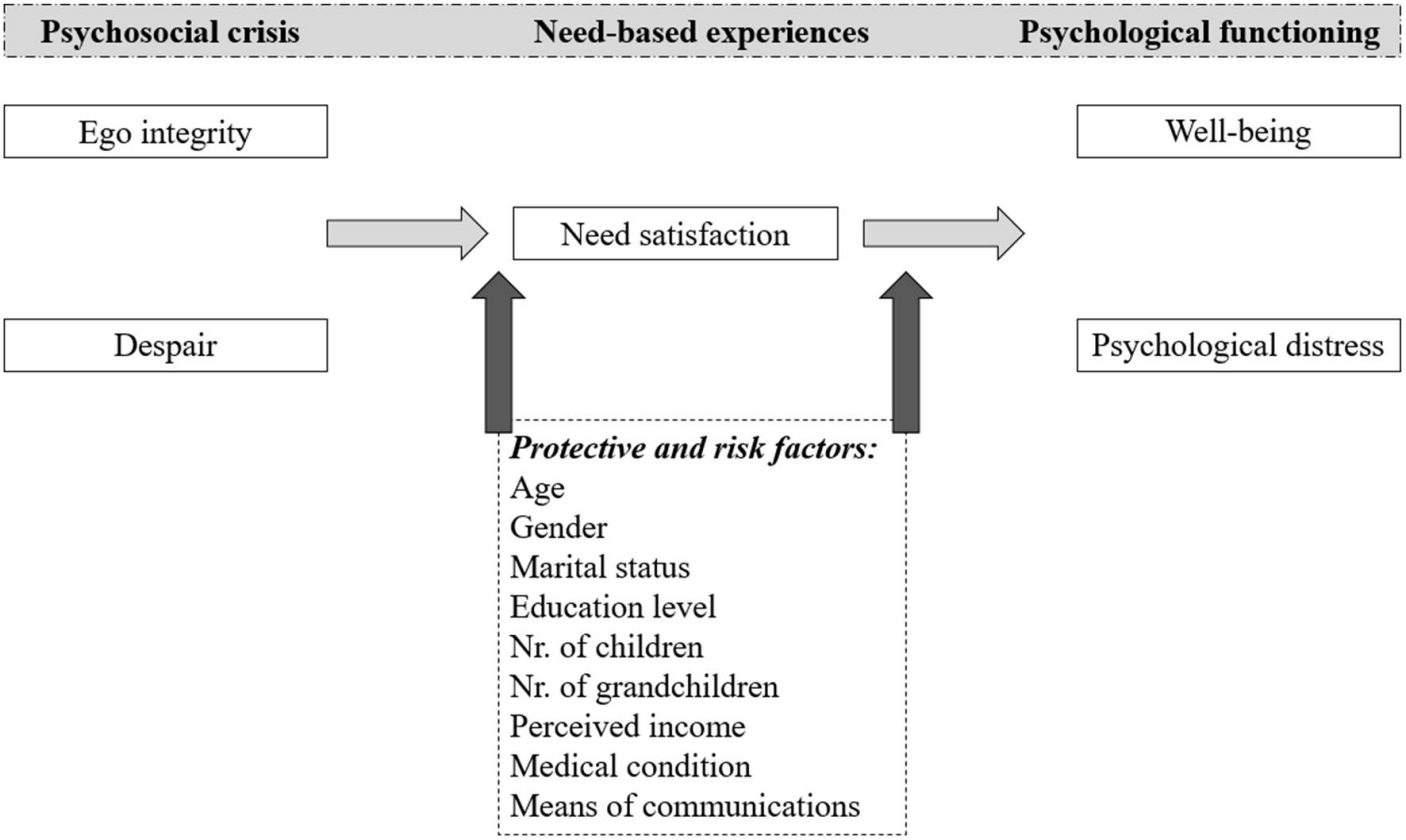
Theoretical Model Displaying the Intervening Role of Need-based Experiences in the Relations from Ego Integrity and Despair to Psychological Functioning, including the Role of Protective and Risk Factors. *Note* The arrows from the protective and risk factors to the study variables represent both main effects (on need-based experiences and psychological functioning) and moderating effects

There exists a lot of heterogeneity among older adults in how they respond to challenges associated with the COVID-19 pandemic (e.g., Bäuerle et al. 2020; Miller 2020). The present research, therefore, sought to better understand late adults’ mental health during a period of crisis through a further cross-fertilization between Erikson’s identity theory and self-determination theory. The theoretical model is displayed in Fig. 1. We hypothesized that ego integrity would serve as a source of resilience to better handle the current COVID-19 crisis, while despair would put older individuals at risk for maladjustment. Drawing on Erikson’s epigenetic principle, older adults who already achieved a sense of ego integrity prior to the COVID-19 outbreak may have more resources available to deal with the uncertainty and threats to one’s basic psychological needs, while the COVID-19 crisis may amplify the vulnerabilities of those who “entered” the crisis high in despair. Specifically, we hypothesized that ego integrity would relate positively to well-being and negatively to psychological distress, whereas an opposite pattern of relations was expected for despair (Hypothesis 1). Further, we hypothesized that need satisfaction would mediate these above-stated relations (Hypothesis 2). Further, we examined in an explorative fashion the role of key risk and protective factors in older adults’ ego integrity, despair, need-based experiences, and psychological functioning, thereby focusing both on possible main and moderating effects of these factors. Finally, we explored the role of each specific psychological need in the relation between ego integrity and despair on the one hand and psychological functioning on the other.

## Method

### Procedure and participants

From March 19 till April 24, 2020, a large and heterogeneous group of Belgian citizens participated in a nation-wide survey. This study was organized to examine citizens’ motivation to abide by measures taken by the government to contain the SARS-CoV-2 virus. Before finishing this online questionnaire, participants were asked whether they were willing to participate in a second survey. Those who agreed to participate a second time with an age over 65 years were contacted for the purposes of the present study between April 24 and May 5. That way, a total sample of 693 older adults (62.1% female, *M*age = 70.06; *SD* = 4.48; range: 65–89 years) was collected. Regarding participants’ marital status, 55.3% was married, 16.5% was divorced, 14.4% was a widow(er), 8.2% was single, and 5.6% was cohabiting. The mean number of children and grandchildren was, respectively, 2.10 and 3.20. The highest level of education obtained was 2.7% primary school or no education, 35.2% high school, 36.8% higher nonuniversity education and 21.1% higher university education (4.2% indicated to have another education not listed in the questionnaire). Almost all participants lived at home (98%). The procedures were approved by the ethics committee of the faculty of psychology and educational sciences of Ghent University (nr. 2020/37).

## Measures

### Risk or protective factors

First, participants were asked to indicate their *age*, *gender*, *marital status*, *education level*, and the *number of children and grandchildren*. With regard to *perceived income*, individuals were asked the following question: “If you think of all the sources of income available to your household, to what extent do you think this income (apart from the corona crisis) is sufficient to make ends meet every month?”. This question was answered on a 4-point Likert scale ranging from 1 (*It is not enough at all. It is very difficult to make ends meet.*) to 4 (*It is certainly sufficient. It works well to make ends meet.*). Participants were also asked to provide information about their *medical condition* by indicating whether they had one of eight medical conditions: respiratory condition (e.g., asthma, tuberculosis), diabetes, heart disease or hypertension, lung disease, liver disease, cancer, disease affecting the immune system, and a disease not specified in the above list. A sum score indicated that participants on average had 0.89 (*SD* = 0.86; range: 0–4) of the listed medical conditions. Individuals also indicated though which *means of communications* they were currently trying to meet their need for social support, by indicating whether they used phone calls, electronic communication (e.g., e-mail, text message), virtual communication (e.g., videocall via Skype or WhatsApp), personal contact, social media (Facebook, Instagram), or another form of communication. A sum score indicated that participants on average employed 3.30 (*SD* = 1.16; range: 1–6) of the listed communication means.

### Ego integrity and despair

To assess ego integrity and despair, we employed a shortened version of the scale developed by Van Hiel and Vansteenkiste (2009). Example items are “I am able to accept the ups and downs of my past life” (ego integrity; 3 items) and “I look back upon my life with a feeling of discontent and regret” (despair; 3 items). Items were rated on a 5-point Likert scale, ranging from 1 (*Completely not true*) to 5 (*Completely true*). Cronbach’s alphas were 0.80 for ego integrity and 0.85 for despair.

### Need satisfaction

We assessed current need-based experiences with a shortened version of the Basic Psychological Need Satisfaction and Need Frustration scale (BPNSNF; Chen et al. 2015). Example items are “I feel a sense of choice and freedom in the things I undertake” (autonomy satisfaction), “I am confident that I can do things well” (competence satisfaction), and “I feel connected to people who care about me and who I also care about” (relatedness satisfaction). Items were rated on a 5-point Likert scale, ranging from 1 (*Completely not true*) to 5 (*Completely true*). Given the high correlation between need satisfaction (six items) and need frustration (six items) (*r* = −0.73, *p* < 0.001) and results of an exploratory factor analysis showing all 12 items to load on one factor, we created a composite score based on the need satisfaction items and the reversed need frustration items. Cronbach’s alpha was 0.88 for this composite score of need satisfaction. In our supplementary analyses, we employed composite scores of autonomy (*α* = 0.78), competence (*α* = 0.80), and relatedness (*α* = 0.76) satisfaction.

### Well-being

To measure life satisfaction and vitality as experienced during the past week, the most face valid items of the Satisfaction with Life Scale (Pavot and Diener 1993) and the Subjective Vitality Scale (Ryan and Frederick 1997), respectively, were selected. Participants were asked to what extent they were satisfied with their life (i.e., life satisfaction) and felt lively (i.e., vitality) during the past week, using a scale going from 1 (*Seldom or never, less than 1 day*) to 4 (*Mostly or all the time, 5 to 7 days*).

### Psychological distress

Depressive symptoms were assessed via a shortened version of the Center for Epidemiological Studies-Depression scale (CES-D; Radloff 1977) which has been used in previous research (Van Hiel and Vansteenkiste 2009), whereas anxiety symptoms were assessed by means of the short form of the State Trait Anxiety Inventory (STAI; Marteau and Bekker 1992), added with the most face valid item of the long form of the STAI (i.e., “I feel anxious”). Following the item stem (i.e., “During the past week”), participants rated six items (e.g., “I felt sad”) relating to depressive symptoms and five items (e.g., “I felt tense”) referring to anxiety symptoms on a scale ranging from 1 (*Seldom or never, less than 1 day*) to 4 (*Mostly or all the time, 5 to 7 days*). Loneliness as experienced during the past week was assessed with four items (e.g., “During the past week, how often did you feel that you had a lack of companionship”) of the UCLA Loneliness Scale (Russell, 1996). Items were rated on a scale ranging from 1 (*Never*) to 5 (*Always*). Cronbach’s alpha was 0.81 for depressive symptoms, 0.81 for anxiety symptoms, and 0.74 for loneliness.

### Plan of analyses

To examine the main hypotheses, we employed structural equation modeling (SEM) with latent variables using MPlus 8.3 (Muthén and Muthén 1998-2017) through a robust maximum-likelihood approach. Latent variables were represented by parcels, where we combined stronger loading items with weaker loading items according to the recommended item-to-construct balance method (Landis et al., 2000). All outcomes were represented by three parcels, except life satisfaction and vitality (which were assessed with only one item). Parcels concerning need satisfaction were created by averaging the items per need (e.g., the four items concerning relatedness satisfaction and the reversed items of relatedness frustration were averaged as to create one parcel for the latent construct of need satisfaction). The individual items concerning ego integrity and despair, which were both assessed with three items, represented the indicators of these latent variables. Several indices were employed to evaluate the fit of these path models, namely the Chi-square test, the Comparative Fit Index (CFI), the Standardized Root Mean square Residual (SRMR), and the Root Mean Square Error of Approximation (RMSEA). Model fit was determined to be acceptable when the *χ*^2^/d*f* ratio was 2 or below and by CFI values of 0.95 or above, SRMR values of 0.08 or below, and RMSEA values of 0.06 or below (Hu and Bentler 1999; Kline 2005). Only 1.36% of the data was missing. These missing data were missing completely at random, as the normed *χ*^2^/d*f* (435.325/247) was 1.76 (i.e., smaller than the recommended cutoff of 2; Ullman 2001). The use of the full information maximum likelihood (FIML) procedure was therefore appropriate to estimate missing data (Schafer and Graham 2002). We used bootstrapping (1000 draws) to test the significance of indirect effects (Preacher and Hayes 2008). Finally, we calculated the percentage of the relation between the predictor (i.e., ego integrity or despair) and the outcomes that could be explained by the mediator (i.e., need satisfaction) thereby employing the formula (*a*-path * *b*-path)/*c*-path.

## Results

### Descriptive statistics and preliminary analyses

Descriptive statistics and bivariate Pearson correlations among the measured variables can be found in Table 1. Further, results of an ANOVA showed that women reported a lower level of life satisfaction (*F*(1,691) = 6.90, *p* = 0.01) and vitality (*F*(1,691) = 4.64, *p* = 0.03) and a higher level of depressive (*F*(1,691) = 16.19, *p* < 0.001) and anxiety (*F*(1,691) = 9.82, *p* = 0.002) symptoms than men. Findings of a second ANOVA showed that marital status had an effect on all study variables, except for need satisfaction. Overall, these findings indicated that married (and to a lesser extent cohabiting) participants displayed better mental health than individuals who were divorced, single, or widowed.

**Table 1:** Descriptives of and correlations between the study variables

		*M*	*SD*	1	2	3	4	5	6	7	8	9	10	11	12	13	14
1	Age	70.06	4.48	–													
2	Education level	4.86	1.33	.02	–												
3	No. of children	2.10	1.50	.17^***^	.01	–											
4	No. of grandchildren	3.20	3.02	.20^***^	.09^*^	.55^***^	–										
5	Perceived income	3.24	0.76	.06	.27^***^	.02	.03	–									
6	Medical conditions	0.89	0.86	.10^*^	−.04	−.01	−.02	−.11^**^	–								
7	Communication	3.30	1.16	-.05	.06	.02	.13^**^	.11^**^	.01	–							
8	Ego integrity	3.96	0.74	.09^*^	.06	.06	.06	.17^***^	−.01	.13^**^	–						
9	Despair	2.13	0.98	-.09^*^	−.10^*^	−.04	−.11^**^	−.25^***^	.12^**^	−.16^***^	−.49^***^	–					
10	Need satisfaction	4.01	0.62	.05	.02	.06	.09^*^	.13^**^	−.09^*^	.15^***^	.40^***^	−.50^***^	–				
11	Life satisfaction	3.20	0.98	.03	.09^*^	.09^*^	.12^**^	.21^***^	−.01	.12^**^	.32^***^	−.36^***^	.47^***^	–			
12	Vitality	2.91	0.99	.01	.05	.10^**^	.12^**^	.17^***^	−.08^*^	.11^**^	.27^***^	−.32^***^	.49^***^	.69^***^	–		
13	Depressive symptoms	1.46	0.51	−.05	−.11^**^	−.03	−.08^*^	−.26^***^	.11^**^	−.07	−.37^***^	.40^***^	−.54^***^	−.59^***^	−.59^***^	–	
14	Anxiety symptoms	2.13	0.66	−.02	−.11^**^	−.05	−.12^**^	−.27^***^	.05	−.07	−.39^***^	.38^***^	−.49^***^	−.68^***^	−.65^***^	.75^***^	–
15	Loneliness	2.64	0.89	−.06	−.02	−.04	−.07	−.18^***^	.05	−.16^***^	−.25^***^	.34^***^	−.58^***^	−.49^***^	−.51^***^	.60^***^	.51^***^

*p < .05; **p < .01; ***p < .001

### Primary analyses

#### The relation from ego integrity and despair to psychological functioning

In a first model, we entered ego integrity and despair as predictors of well-being indicators (i.e., life satisfaction and vitality) and psychological distress indicators (i.e., depressive symptoms, anxiety symptoms, and loneliness). This model had an adequate fit; *χ*^2^/d*f* = 3.23; CFI = 0.95; SRMR = 0.04; RMSEA = 0.06. Results showed that ego integrity and despair both related to all indicators of well-being and psychological distress in the expected direction (β ranging between 0.14 and 0.35 for the positive effects and between −0.25 and −0.29 for the negative effects, *p*s < 0.01), except for a nonsignificant relation between ego integrity and loneliness (*β* = −0.09, *p* = 0.11). Results from five Satorra–Bentler scaled Chi-square difference tests showed that the effects of despair on individuals’ psychological functioning were stronger than the effects of ego integrity; Δ*χ*(1) ranging between 31.05 and 63.17, *p*s < 0.001. Ego integrity and despair were moderately and negatively correlated (*r* = −0.60, *p* < 0.001). All outcomes were significantly related to one another. That is, the indicators of psychological distress were strongly positively correlated with one another (*r* ranging between 0.55 and 0.83), whereas these were negatively correlated with the indicators of well-being (*r* ranging between −0.46 and −0.86). Finally, vitality and life satisfaction were positively correlated (*r* = 0.63).

#### The mediating role of need-based experiences

**Fig. 2:**
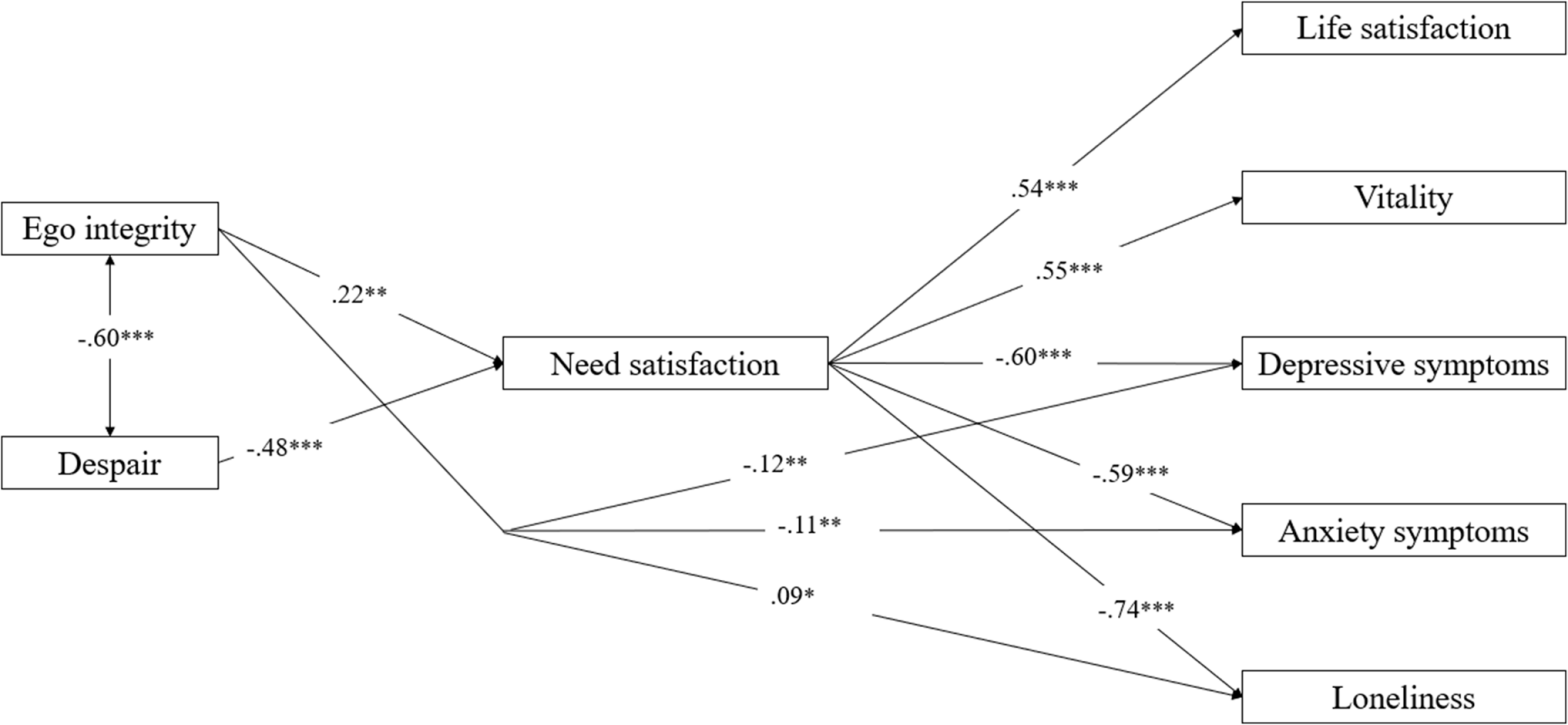
Structural Model Depicting the Intervening Role of Need-based Experiences in the Relations from Ego Integrity and Despair to Psychological Functioning. *Note* Correlations between the outcomes were allowed but are not shown for reasons of clarity. *p < .05; **p < .01; ***p < .001

Building on the previous model, in a second model we added need satisfaction as a mediating variable between ego integrity and despair on the one hand and the indicators of well-being and psychological distress on the other. Additionally, three significant direct effects from ego integrity to depressive symptoms, anxiety symptoms, and loneliness were added. This model had a good fit; *χ*^2^/d*f* = 3.44; CFI = 0.94; SRMR = 0.05; RMSEA = 0.06. As displayed in Fig. 2, ego integrity related positively to need satisfaction which, in turn, related to a higher level of well-being and a lower level of psychological distress, with despair showing an opposite pattern of relations. Results from a Satorra–Bentler scaled Chi-square difference test further showed that despair was more strongly related to need satisfaction than ego integrity; Δχ(1) = 85.08, *p* < 0.001. Further, ego integrity related also directly and negatively to depressive symptoms and to anxiety symptoms. Surprisingly, ego integrity related positively to loneliness. 1 All indirect effects were found to be significant. That is, ego integrity related through need satisfaction to the indicators of well-being (95% CI ranging between 0.044 and 0.199) and psychological distress (95% CI ranging between −0.266 and −0.048). Similarly, despair related via need satisfaction to well-being (95% CI ranging between −0.339 and −0.186) and psychological distress (95% CI ranging between 0.203 and 0.451). With respect to ego integrity, need satisfaction explained a substantial part of its effect on life satisfaction (56.2%), vitality (90.8%), depressive symptoms (54.5%), anxiety (45.9%), and loneliness (100%). The mediating role of need satisfaction was even stronger for the effects of despair on life satisfaction (85.2%), vitality (100%), depressive symptoms (85.8%), anxiety (96.9%), and loneliness (100%). Finally, correlations between the outcomes were similar compared to the previous model but became somewhat smaller.

#### The role of risk and protective factors

Finally, we aimed to examine the role of the risk or protective factors by first adding these factors as main effects to the model with respect to both the mediator (i.e., need satisfaction) and the outcomes. This model had an adequate fit; *χ*^2^/d*f* = 2.91; CFI = 0.91; SRMR = 0.07; RMSEA = 0.05. Associations between the main study variables (i.e., ego integrity, despair, need satisfaction, and outcomes) were equivalent to the previous model. With respect to the risk or protective factors, 12 main effects were found to be significant which are displayed in Table 2. Specifically, a higher level of perceived income was related to more life satisfaction and less depressive symptoms, anxiety and loneliness. With respect to gender, female participants reported less life satisfaction and a higher level of depressive symptoms. The more participants reported using different means of communication, the lower their level of loneliness. Individuals who were divorced, single, and widowed had a higher level of depressive symptoms than their counterparts, with widowed individuals also experiencing less vitality. Finally, having more grandchildren related to less anxiety.

**Table 2:** Main effects of the risk or protective factors

	Need satisfaction	Life satisfaction	Vitality	Depressive symptoms	Anxiety symptoms	Loneliness
	*β* (*p* value)	*β* (*p* value)	*β* (*p* value)	*β* (*p* value)	*β* (*p* value)	*β* (*p* value)
Gender	–	–.09 (.01)	–	.08 (.02)	–	–
Marital status	–	–	Widowed: –.12 (.04)	Divorced: .15 (.02) Single: .15 (.01) Widowed: .21 (.001)	–	–
No. of grandchildren	–	–	–	–	−.09 (.02)	–
Perceived income	–	.14 (< .001)	–	−.12 (.003)	−.19 (< .001)	−.08 (.04)
Communication	–	–	–	–	–	−.08 (.04)

**Fig. 3:**
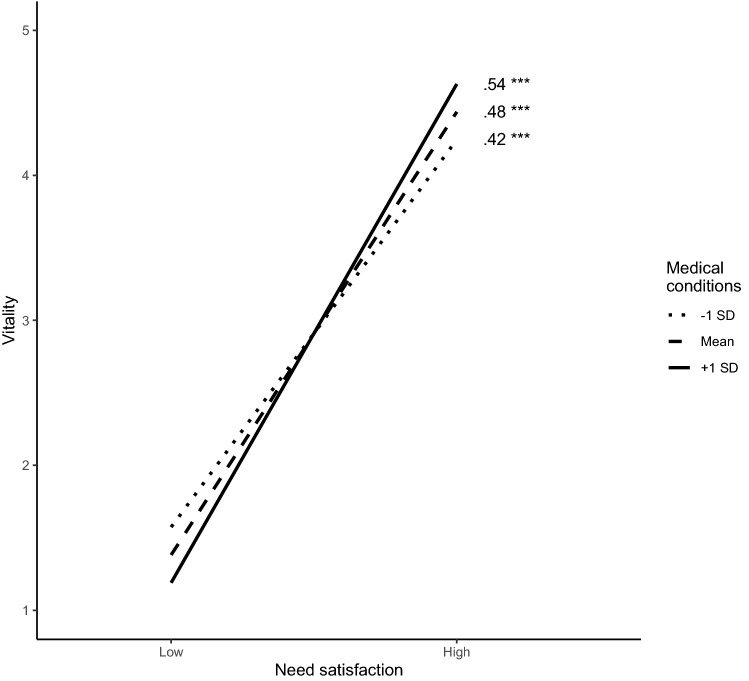
Graphical Representation of the Interaction Effect, including Standardized Simple Slopes

Subsequently, we examined the possible moderating role of the risk or protective factors by adding the interaction term between each of these factors and the predictors (i.e., ego integrity, despair, and need satisfaction) one by one to the mediation model. Only one interaction was found to be significant, from which the simple slope analysis and moderation is plotted in Fig. 3. This analysis showed that more need satisfaction related to more vitality, with stronger effects among those with more medical conditions (*β* = 0.07, *p* = 0.02; 95% CI [0.012, −0.117]). However, this effect was small, with the explained variance in vitality (*R*^2^ = 0.31) remaining the same when adding this interaction effect to the model.

### Supplementary analyses

**Fig. 4:**
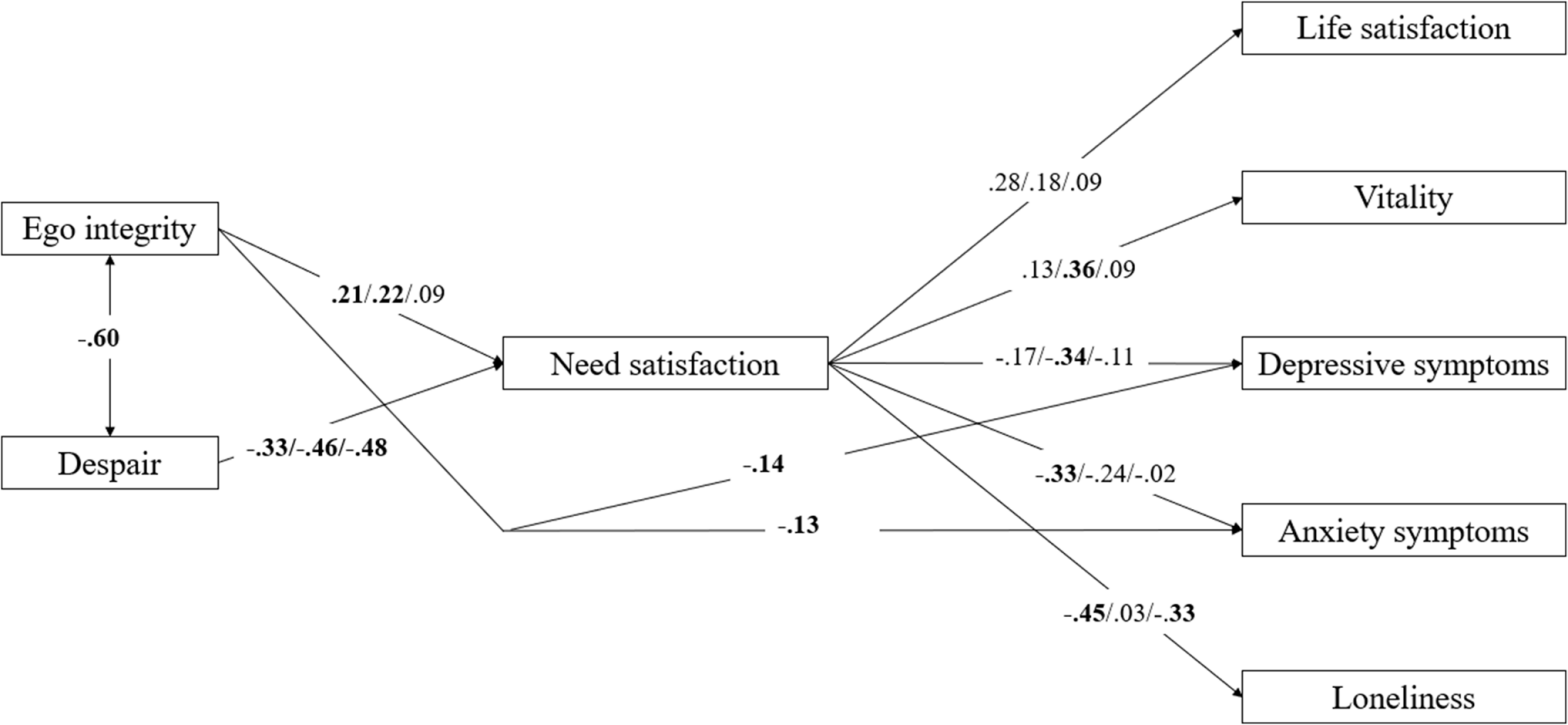
Structural Model Depicting the Intervening Role of Specific Need-based Experiences in the Relations from Ego Integrity and Despair to Psychological Functioning. *Note* Standardized coefficients concerning the satisfaction of, respectively, autonomy, competence, and relatedness are reported sequentially and separated by a slash. Significant standardized coefficients are indicated in bold. Correlations between the outcomes were allowed but are not shown for reasons of clarity

In an explorative fashion, we examined what the mediating role was of each specific need in the relation from ego integrity and despair to the outcomes. By doing so, we built on Model 2, thereby replacing overall need satisfaction with the three latent constructs of autonomy satisfaction, competence satisfaction, and relatedness satisfaction. This model had an adequate fit; *χ*^2^/d*f* = 2.81; CFI = 0.92; SRMR = 0.05; RMSEA = 0.05. As displayed in Fig. 4, ego integrity related to the satisfaction of autonomy and competence, but not relatedness, whereas despair related to all three needs satisfaction. The relations from each need to the outcomes were more specific. That is, vitality was only predicted by competence satisfaction, whereas none of the needs uniquely predicted life satisfaction. Regarding psychological distress, only competence satisfaction related uniquely to depressive symptoms, only autonomy satisfaction related uniquely to anxiety, and both autonomy and relatedness satisfaction related to loneliness. Similar to Model 2, ego integrity related also directly to depressive symptoms and to anxiety (but not to loneliness anymore). Autonomy satisfaction was strongly related to competence (*r* = 0.79) and relatedness (*r* = 0.58) satisfaction, whereas the relation between competence and relatedness satisfaction was moderately strong (*r* = 0.47), all *p*s < 0.001. Relations between ego integrity and despair and between the outcomes were similar to the previous models. Four indirect effects were found to be significant. That is, ego integrity related through autonomy satisfaction (explaining 97.4%) to loneliness (95% CI [−0.181., −0.004]). Further, despair related via competence satisfaction (explaining 79.3%) to vitality (95% CI [−0.293, −0.033]). Finally, despair related through autonomy (95% CI [0.016, 0.285]) and relatedness (95% CI [0.078, 0.241]) satisfaction to loneliness, with these mediators explaining, respectively, 45.2% and 44.4% of the effect from despair to loneliness.

## Discussion

The COVID-19 pandemic has posed an immediate threat to individuals’ health, while disturbing and restricting individuals’ daily lives. Extant research indicates that older adults should be considered as a very heterogeneous group, with some older adults reporting decreased well-being or even increased psychological distress (Miller 2020) and others experiencing similar or higher levels of well-being during this pandemic (Kivi et al. 2020) compared to before the pandemic. The overall aim of this study was, therefore, to examine what factors increase or threaten older adults’ ability to adapt to this crisis. In doing so, we sought for a cross-fertilization between two well-established theories of human development, namely Erikson’s personality theory and self-determination theory.

Previous studies have consistently shown the benefits of ego integrity for older adults’ psychological functioning. Few studies, however, focused on the unique role of despair, often creating a composite score of ego integrity versus despair. Herein, we considered ego integrity as a source of resilience and despair as a vulnerability factor to “enter” the COVID-19 outbreak. Current findings confirm the separate contributions of ego integrity and despair, with both ego integrity and despair uniquely relating to life satisfaction, vitality, depressive symptoms, and anxiety symptoms, yet in opposite ways. Note, however, that only despair related to loneliness. Interestingly, effects of despair were somewhat stronger than the effects of ego integrity. Perhaps despairing over one’s past life is especially detrimental during stressful times of social isolation, where reassurance from others is not always available and people might end up ruminating excessively over personal regrets.

Despite studies showing the importance of ego integrity and despair for older adults’ psychological functioning, less is known about the underlying mechanisms behind these relations. In line with SDT and with previous studies showing ego integrity to be related to more indirect measures of need-based experiences (e.g., James and Zarrett 2006), we found that the effects of ego integrity and despair on well-being and psychological distress were partially mediated by the experience of need satisfaction (although three direct effects of ego integrity remained significant). This indicates that when older adults are able to come to terms with their past, they experience more need satisfaction during more difficult moments, as those encountered during the COVID-19 crisis. Presumably, those who have already achieved a sense of ego integrity experience greater need satisfaction from their current activities, either because they may self-select themselves into more need-satisfying activities, because they perceive situations in a more positive, need-conducive light, or because they may elicit more need-supportive responses from those around them. When focusing specifically on the satisfaction of each of the three needs, the relations from ego integrity and despair to individuals’ need satisfaction were largely replicated (with the exception of a nonsignificant relation between ego integrity and relatedness). However, the relations between need satisfaction and the outcomes were found to be more need-specific, with for instance only competence satisfaction being related to depressive symptoms. As the items concerning need satisfaction and reversed need frustration were shown to load on one factor and because of the high correlations between the satisfaction of each of the three needs, the current need-specific findings should be interpreted with caution. Satisfaction of one need (e.g., competence) often goes hand-in-hand with another need’s satisfaction (e.g., autonomy). Looking at unique effects of each need disregards, therefore, an essential part of the experience of need satisfaction. Further, given that our assessment of need-based experiences focused on momentary feelings, a high correlation between each of the needs is very likely. That is, although individuals can in general state that they experience a high level of autonomy satisfaction (e.g., in their job) and a low level of relatedness (e.g., in their romantic relationship), experiencing such a discrepancy between the satisfaction of different needs at one specific moment is more rare. More research on possible sources of discrepancies between different needs satisfaction (for instance, by using a person-centered perspective; Tóth-Király et al. 2020) is, however, needed to shed more light on this issue.

Another aim of this study was to examine what factors might put older adults more at risk for maladaptive functioning during the COVID-19 crisis, or, in contrast, what factors could foster a more adaptive functioning during these challenging times. Specifically, we focused on the role of age, gender, marital status, education level, number of children, number of grandchildren, perceived income, sum of the medical conditions, and sum of means of communications, thereby examining both their main effects on (mal) adaptation and their possible moderating role. We found that having a higher perceived income, being male, having more grandchildren, and not being widowed, single or divorced were protective factors, relating to a higher level of well-being and a lower level of psychological distress. These results are in line with previous studies showing the positive effects of income (Lukaschek et al. 2017), being a grandparent and male (Tanskanen et al. 2019), and being in a romantic partnership (Carr and Springer 2010) on older adults’ well-being. Surprisingly, although previous studies among older adults have shown age (e.g., Hansen 2020) and poor health (e.g., Steptoe et al. 2015) to be negatively related to psychological functioning, and education to be positively related to psychological functioning (e.g., Wiesmann and Hannich 2008), our main findings showed no such effects. The lack of effects of age and health could be due to the rather limited number of very old individuals (aged over 80) and of individuals with significant poor health in the current sample. Further, the current findings also seem to indicate that psychological factors such as need satisfaction, ego integrity, and despair are more important predictors of individuals’ psychological functioning than merely their age, health condition, or educational level. For future studies, it would also be interesting to examine the possible intervening role of these psychological factors in the relation from, for instance, health condition to well-being. To illustrate, individuals with a poor health are likely to experience more difficulties in maintaining their relationships (i.e., need for relatedness), experiencing choice and freedom in daily life (i.e., need for autonomy), and in accomplishing personal goals (i.e., need for competence), resulting in less experienced well-being.

Interestingly, we also found that older adults who used more different means of communication (e.g., phone calls, electronic communication such as text messages, and virtual communication including Skype or WhatsApp) reported a lower level of loneliness. The use of different communication means is especially important in old age, as older adults often face increasingly communication problems due to, for instance, cognitive decline or decreasing physical health. For instance, a large survey among adults aged 65 years or more showed that 42% reported hearing problems, 26% had writing problems, and 7% had problems using the telephone (Hoffman et al. 2005). Current findings seem to indicate that older adults who are more able to adapt their means of communication to their communication capabilities and needs thrive more, although more research on this issue is needed. Further, we found little evidence for the moderating role of these assessed factors, with only one interaction being significant. That is, participants who reported a higher level of medical conditions were more affected by need satisfaction, reporting lower vitality when experiencing low need satisfaction and higher vitality when experiencing high need satisfaction. It is important to note, however, that this interaction effect was rather weak (explaining no additional variance in vitality) and only indicated a difference in strength of this relation, with the main effect of need satisfaction on vitality being significant across all participants (irrespective of level of medical conditions).

### Limitations and future challenges

This study had several important strengths, including the assessment of older adults’ well-being in a historical time period (i.e., the COVID-19 pandemic), the use of a large sample, including indicators of both adaptive and maladaptive psychological functioning, and the cross-fertilization of two well-established theories on human development (i.e., Erikson’s personality theory and SDT). Nonetheless, the study also had a number of important limitations.

First, this study used a cross-sectional design, thereby precluding the examination of possible reciprocal effects between, for instance, despair and psychological distress. It could be the case that current negative feelings invoke more despair and regret over the past due to making negative memories from the past more salient. Future research employing more dynamic designs (e.g., longitudinal, diary) would be crucial in gaining more insight into these possible reciprocal associations. Relatedly, our study did not include a pre-COVID-19 assessment, thereby limiting the examination of possible changes in the study constructs due to this pandemic.

On a methodological level, this study was limited by the use of single items for two of the outcomes (i.e., life satisfaction and vitality) and the employment of self-reports, which can cause same-source bias, shared method variance, and retrospective bias. Although the use of self-reports for the processes of ego integrity, despair, and need-based experiences is justifiable given the highly internal nature of these constructs, it would be informative to also have other informants report on the older adult’s psychological functioning in future studies. Further, in line with other studies done in the context of COVID-19 (Losada-Baltar et al. 2020), our sample consisted of older adults who were able to use online technologies which may limit the generalizability of our sample. Indeed, research has shown that respondents who prefer to use a paper (instead of online) questionnaire, were more likely to be female, retired, single, and to report a lower level of education, higher levels of depression and lower self-reported health (Kelfve et al. 2020). Additionally, although we examined a broad set of potential risk and protective factors, there could be other factors that might be important in understanding older adults’ coping with the COVID-19 pandemic including for instance religious affiliation, ethnicity, or geographical location.

Almost all participants included in this study lived at home, restricting the generalizability of the current findings. As stated by Gardner et al. (2020), older adults living in care facilities during this crisis have an increased risk to experience social isolation and potentially even abuse and neglect compared with older adults living at home. Previous research stemming from before the pandemic has also shown older adults residing in hostels (instead of self-care apartments) to experience less ego integrity (assessed as the degree of accepting the past) (Rylands and Rickwood 2001). It would therefore be important to replicate the current findings also among older populations living in care facilities.

## Conclusion

Accepting and integrating past events into one’s self plays a critical role in older adults’ current functioning and well-being (e.g., Derdaele et al. 2019). The present study showed that a successful resolution of ego integrity versus despair is crucial for older adults’ psychological functioning, even (and perhaps especially) during a pandemic crisis. Adding to previous literature by shedding light on mechanisms and bridging the gap between two well-established theories in human development, current findings suggest that need-based experiences are crucial intervening processes in the associations of ego integrity and despair with mental health. Finally, results showed that some older adults (e.g., those with a higher perceived income) are more resilient in coping with this crisis, whereas others (e.g., those being widowed) are more vulnerable during these challenging times.

## Footnotes

1 The positive relation between ego integrity and loneliness should be interpreted with caution, given that the zero-order correlation indicated a negative relation (i.e., *r* = -.25, *p* < .001) and the results of Model 1 showed a nonsignificant relation (i.e., β = -.09, *p* = .11) between these construct. The significant relation found in the structural model suggests a suppressor effect where the addition of a predictor (in this case, need satisfaction) increases the predictive power of another variable (in this case, ego integrity).
